# Quadriceps Corticospinal and Intracortical Excitability Assessment Using Transcranial Magnetic Stimulation: A Test–Retest Reliability Study

**DOI:** 10.3390/neurosci7030069

**Published:** 2026-06-13

**Authors:** Liam C. Tapsell, Molly E. Coventry, Colin Sylvester, Casey Whife, Myles C. Murphy

**Affiliations:** 1 Nutrition and Health Innovation Research Institute, School of Medical and Health Sciences, Edith Cowan University, Joondalup, WA 6027, Australia; l.tapsell@ecu.edu.au (L.C.T.); m.murphy@ecu.edu.au (M.C.M.); 2 Medical and High-Performance Department, Fremantle Football Club, Cockburn Central, WA 6164, Australia; 3 Medical and High-Performance Department, West Coast Eagles Football Club, Lathlain, WA 6100, Australia; 4 Institute for Health Research, School of Health Sciences, The University of Notre Dame Australia, Fremantle, WA 6160, Australia

**Keywords:** lower limb, motor evoked potential, rectus femoris, corticospinal excitability, intracortical inhibition, intracortical facilitation, motor threshold

## Abstract

Objective: Evaluate the test–retest reliability of quadriceps corticospinal excitability and intracortical excitability using transcranial magnetic stimulation (TMS). Design: A test–retest observational cohort study. Methods: Twelve healthy adults attended two laboratory sessions, seated with their knee at 90 degrees and fitted with electrodes on the rectus femoris (RF), vastus lateralis (VL) and vastus medialis (VM). TMS was used to assess the active motor threshold (AMT), motor evoked potential (MEP) amplitude, short-interval intracortical inhibition (SICI) and intracortical facilitation (ICF). Individual results were calculated as the mean and median of the 10 trials of each measure for MEP, SICI and ICF. Intraclass correlation coefficients were calculated. Results: All muscles showed good or excellent reliability for the mean and median measures of the MEP amplitude (ICC ≥ 0.820) as well as the AMT of the RF (ICC = 0.991). SICI showed good reliability in the mean and median measures of the RF and the mean measure of the VL (ICC ≥ 0.809), moderate reliability in both measures of the VM (ICC ≥ 0.655) and was not significant for the median measure of the VL (ICC = 0.513). ICF showed excellent reliability in the mean measure of each muscle and median measure of the RF (ICC ≥ 0.906), with good reliability in the median measure of the VL (ICC = 0.888) and moderate reliability in the median measure of the VM (ICC = 0.719). Conclusion: The mean of an individual’s quadriceps corticospinal excitability and intracortical excitability have good or excellent reliability for every TMS measure, in every muscle (except SICI of the VM). With the previous reliability of TMS measures mostly investigating the upper limbs, these results offer important context for neurophysiological research in the quadriceps.

## 1. Introduction

Measures of corticospinal excitability are assessed using multiple single-pulse transcranial magnetic stimulation (TMS) methods [1], with muscle responses measured via electromyography (EMG). Common measures of corticospinal excitability include motor evoked potentials (MEPs, the amplitude of EMG signal generated by suprathreshold stimulation) and the active motor threshold (AMT, defined as the minimum stimulator intensity required to generate an MEP in at least half of attempts) [2]. Higher corticospinal excitability is indicated by lower active motor thresholds and larger MEPs and vice versa.

Measures of intracortical excitability are assessed using paired-pulse TMS. In this paradigm, the suprathreshold (test) stimulus is preceded by a conditioning stimulus, which is typically less than the AMT [3]. Importantly, the conditioning stimulus should not elicit an MEP but engages intracortical processes that alter the amplitude of the MEP elicited by the second pulse test stimulus. As an example, short-interval intracortical inhibition (SICI) can be evaluated with a conditioning stimulus of an 80% AMT, a test stimulus of a 120% AMT, and an interstimulus interval (ISI) of 3 ms [4]. Intracortical facilitation (ICF) can be evaluated with a conditioning stimulus of an 80% AMT, a test stimulus of a 120% AMT and an ISI of 12 ms (e.g., the conditioning pulse is performed 12 ms prior to the test pulse) [4].

The assessment of corticospinal and intracortical excitability is increasingly valued in research exploring the mechanisms that influence and drive musculoskeletal pain, injury and recovery [5]. For example, in hip osteoarthritis, it was found that higher pain levels were associated with lower ICF levels [5]. In knee osteoarthritis, greater pain was associated with lower levels of ICF as well as higher resting motor thresholds (RMTs) [6].

Despite the increasingly common use of TMS to quantify corticospinal and intracortical excitability in lower-limb musculoskeletal conditions, only a few studies have assessed the reliability of these methods in the quadriceps [7,8,9] and hamstrings [10], with none in the triceps surae. Specifically, MEP size, AMT silent periods and short- and long-interval intracortical inhibition (LICI) have been evaluated in two quadricep muscles (vastus lateralis and rectus femoris), and only SICI, LICI and ICF have been evaluated in the hamstrings. These studies have demonstrated moderate to excellent reliability for repeat measure testing on the same day, between days and for different muscle contraction types (e.g., isometric, concentric and eccentric).

It is well accepted that the MEP amplitude is highly variable within subjects [11]. Therefore, at least 10 measures are typically taken and pooled to represent a single data value [12]. This is typically calculated using the mean or, to avoid instances in which a single MEP excessively alters the result, the median. The use of the mean has shown sufficient reliability for measures of the AMT, MEP, SICI, and ICF in the upper limb [13]. However, the reliability of these two measures has not been compared for lower limb TMS testing. The upper limb has been more frequently evaluated with TMS in research, as testing in the lower limb has additional complexities, such as deeper motor representations and a closer proximity to the contralateral limb’s motor representation [14]. Thus, it is sensible that initial research into TMS reliability has focused on the upper limb. However, the reliability in the upper limb may not be able to be generalised to the lower limb. Furthermore, TMS research in the lower limb commonly uses a different (double-cone) coil than the upper limb, as well as greater intensities, due the depth of the cortical representation [15]. Given these differences in methodology, it is pertinent to assess the reliability of TMS measures in the lower limb specifically. As the quadriceps are commonly investigated in the literature, and are integral to hip and knee movements, we selected the quadriceps to evaluate lower limb reliability.

### Objectives

The primary objective of this study was to evaluate the test–retest reliability of quadriceps corticospinal excitability (AMT and MEP) and intracortical excitability (SICI and ICF) using TMS when corticospinal excitability and intracortical excitability were quantified from median or mean scores.

## 2. Methods

### 2.1. Study Design

This reliability study was nested within a randomised crossover study to evaluate the immediate effects of a novel transcranial direct current stimulation headset. The two sessions performed to evaluate reliability were performed prior to the transcranial direct current stimulation and beyond the maximum reported time for corticospinal and intracortical changes from transcranial direct current stimulation [12] to ensure wash-out.

### 2.2. Methodological Guidelines

This study was informed by, and reported in accordance with, the Strengthening the Reporting of Observational Studies in Epidemiology (STROBE) guideline for reporting observational studies [16].

### 2.3. Participants

We included healthy adults aged over 18 years. Participants were excluded for the following reasons: pregnancy; neurological conditions/illness (including epilepsy/convulsion/seizure); vascular, traumatic, tumoural, infectious, or metabolic lesion of the brain; previous or current implants in their body that may be triggered or heated by an electrical current (e.g., pacemaker, intracranial shunts, artificial cochlea); any metal implanted in their head (e.g., surgical clips, staples, shrapnel); frequent or intense headaches; previous brain trauma or neurosurgical intervention; serious medical complications (e.g., advanced pulmonary, cardiac, liver or kidney disease); currently taking neuropsychotropic drugs (e.g., antiepileptics, neuroleptics, benzodiazepines or antidepressants) or drugs with an effect on neuroplasticity (dopamine, fluoxetine, D-amphetamine, sodium/calcium channel blockers or NMDA receptor antagonists); and sleep deprivation the night before testing.

### 2.4. Setting

**Figure 1:**
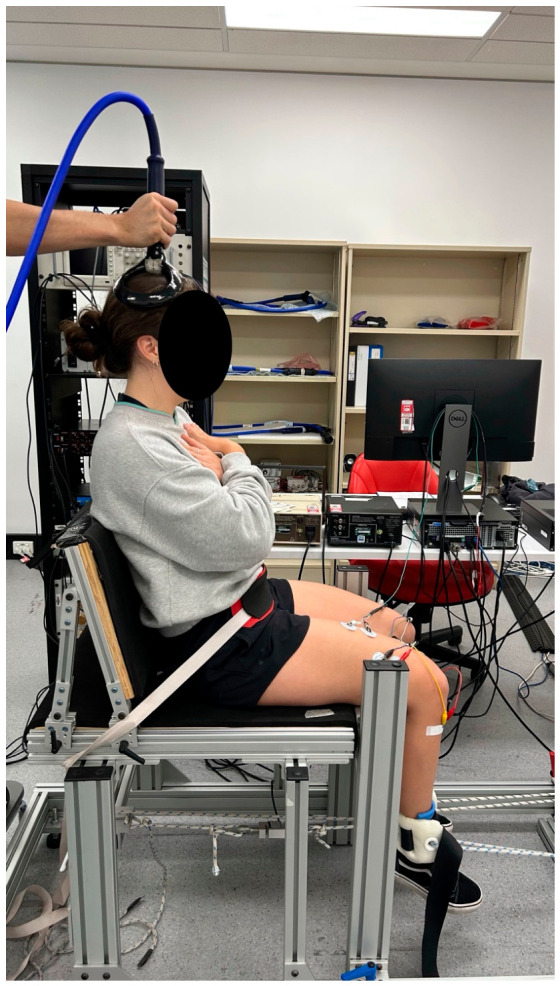
Participant set-up in isometric dynamometer for knee extension and placement of electromyography electrodes and transcranial magnetic stimulation coil.

Participants attended two experimental sessions interspaced by at least 72 h, but less than 7 days, at the neurophysiology laboratory at Edith Cowan University in Joondalup, Western Australia. The time of day that the testing occurred was kept as similar as possible between sessions 1 and 2. Participants were seated in a custom-built chair (80/20 Australia), designed to measure their isometric knee extension strength (Figure 1), which we have described previously [5]. The right lower leg was secured to an UU-K100 100 kg in-line force transducer (DACELL Co., Ltd., Cheongju, Republic of Korea) via a Velcro strap placed approximately 2 cm above the ankle. A rope connects the ankle Velcro strap to an immovable bar behind it, and the tension on the rope is adjusted to increase/decrease the knee joint flexion angle. The backrest of the chair was adjusted to fit each participant’s height and limb length, with the knee positioned at 90° of flexion (confirmed via goniometry). Finally, a belt was secured around the participant’s waist to prevent any movement.

### 2.5. Experimental Procedure

Maximal voluntary isometric contractions (MVICs) of knee extension were recorded at the commencement of each assessment session, which was used to set contraction intensities during TMS testing. Following warm up trials at approximately 25%, 50% and 75% MVICs, participants performed three MVICs for ~3 s each while the assessor (LT) provided strong verbal encouragement, with 60 s rest provided between each attempt. The surface EMG signal was recorded from the vastus medialis (VM), vastus lateralis (VL), and rectus femoris (RF) using bipolar Ag/AgCl self-adhesive electrodes with a 50 mm inter-electrode distance. The skin of the thigh was cleaned with 70% isopropyl alcohol swabs prior to placing the electrodes over the muscles of interest. Recording electrodes were placed as recommended by the surface EMG for a non-invasive assessment of muscles (SENIAM, http://seniam.org/, accessed on 13 April 2026) guidelines. VL, VM and RF electrodes were positioned at 66%, 80% and 50% of the distance between the inguinal crease and the superior border of the patella respectively and were placed parallel to the angulation of the muscle fibres. EMG signals were amplified (×1000), filtered (20 Hz–1 kHz, CED 1902 amplifier, Cambridge Electronic Designs, Cambridge, UK) and digitised at 2 kHz (CED 1401) before being stored on Microsoft Teams for analysis (CED Spike 2 V7.20 software).

### 2.6. Transcranial Magnetic Stimulation

Transcranial magnetic stimulation was used to assess the excitability of the primary motor cortex (M1) for the quadriceps muscles during a 10% MVIC [9]. A 110 mm double-cone coil connected to a BiStim 2002 magnetic stimulator (Magstim Co., Dyfed, UK) was used, with the coil positioned to direct the current in an anterior–posterior direction. A single experienced assessor (LT) held the TMS coil for TMS assessments across all sessions and participants for consistency. The optimal stimulation site (i.e., the ‘hotspot’) for TMS was identified as the site that produced the largest and most consistent MEP of the RF [1]. The RF was selected as the target muscle based on our previous research [5], whereby the RF has the smallest MEP when compared to the VL and VM. Thus, by ensuring the RF had consistent MEPs, we expected consistent responses in the VL and VM. The hotspot location was identified each session and marked on a cap (Original Skull, Under Armour, Baltimore, MD, USA) worn by the participant. The AMT was determined as the lowest possible level of stimulation output required to elicit at least 3 out of 6 MEP responses from the RF that were visually larger than the background EMG during a submaximal isometric contraction at a 10% MVIC. Due to the AMT being used to calculate stimulation intensity in other measures, only assessing the AMT in the RF was appropriate.

The paired-pulse technique involved delivering a test pulse that was preceded by a subthreshold (conditioning) stimulus with a 3 ms inter-stimulus interval for SICI and 12 ms ISI for ICF. The conditioning stimulus and test stimulus intensities were set at 80% and 120% of the AMT (as measured in that session), respectively. Each participant received a total of 10 × MEP_120_ (unconditioned MEP at 120% AMT), 10 × SICI, and 10 × ICF stimuli, with the order in which the pulses were delivered randomised in 5 blocks, each containing stimuli for 2 MEP, 2 SICI, and 2 ICF measures. Each stimulus block was delivered during a 10% knee extension MVIC with a ~30-s duration, followed by 60 s of rest. Six stimuli were delivered while the participants were instructed to maintain the force output at 10% MVIC, with visual feedback of force provided on a computer screen in front of them. The TMS coil was removed between blocks to avoid movement of the marked scalp cap. Single- and paired-pulse MEP sizes were quantified by measuring the peak-to-peak amplitude (mV). The MEP amplitudes from unconditioned, SICI and ICF protocols for each participant were then converted to a mean and median, with the reliability of both methods calculated. Unconditioned MEPs were reported as the raw value. SICI and ICF were expressed as a ratio (amplitude of conditioned MEP/amplitude of unconditioned MEP).

### 2.7. Variables

#### 2.7.1. Demographics

We collected the following demographic data via Qualtrics survey software (Qualtrics, Provo, UT, USA), which was completed prior to commencing the physical assessment: age (years), sex (male; female; intersex), race (White; Black or African American; American Indian or Alaska Native; Asian; Native Hawaiian or Other Pacific Islander), height (cm) and body mass (kg). Body mass index (BMI) was calculated as [body mass (kg)/height (m)^2^].

#### 2.7.2. Quadriceps Strength

The MVIC was measured in newtons from in-line dynamometry.

#### 2.7.3. Motor Cortex Excitability

Single-pulse TMS was used to measure AMT (percentage of maximal stimulator output, %MSO) and peak-to-peak MEP amplitudes (mV).

#### 2.7.4. Intracortical Excitability

Paired-pulse TMS was used to measure SICI (size of conditioned MEP as a percentage of unconditioned MEP) and ICF (size of conditioned MEP as a percentage of unconditioned MEP).

### 2.8. Statistical Analysis

Demographic data were described as the mean (SD, standard deviation), with ranges given where appropriate. For the assessment of reliability, we calculated the intraclass correlation coefficient (ICC) comparing participants’ results in their first and second sessions. In addition, the standard error of measurement (SEM) and 95% ICC confidence intervals were calculated. All statistical analyses were completed using Jamovi (version 2.3, The Jamovi Project). Each of these measures were calculated by comparing participants’ results from their first session to their second session. As only a single value for the AMT was recorded, the ICC was selected based on the two-way mixed effects, absolute agreement, and single measurement. As the 10 values for each the MEP, SICI, and ICF were used to calculate participant mean or median values, the ICC was also selected based on the two-way mixed effects, absolute agreement, and single measurement. We evaluated the reliability of our procedure for each outcome of interest (AMT, MEP, SICI and ICF) and for each muscle of interest (VM, RF and VL). Finally, for MEP, SICI, and ICF, we assessed the reliability by taking the mean of the 10 trials, as well as by taking the median of the 10 trials. ICC estimates less than 0.5 indicate poor reliability, between 0.5 and 0.75 indicate moderate reliability, between 0.75 and 0.9 indicate good reliability and values greater than 0.9 indicate excellent reliability [17]. All TMS data are presented in scatter plots. Statistical significance was accepted when *p* < 0.05.

## 3. Results

### 3.1. Participant Characteristics

Twelve healthy participants (eight male, four female) were included, with no drop out between testing sessions. Participants self-reported their race as White (n = 9), Asian and White (n = 1), Asian (n = 1) and Hispanic/Latino (n = 1). Participants were a mean age of 38 (SD = 15, range = 22 to 65) years, with a height of 175 (SD = 10, range = 160 to 186) cm, a body mass of 75 (SD = 17, range = 52 to 110) kg and a BMI of 25 (SD = 3, range = 20 to 33) kg/m^2^, with MVICs in session one of 320 (SD = 104, range = 202 to 480) N and MVICs in session two of 357 (SD = 109, range = 230 to 544) N. Participants’ neurophysiological results are shown in Table 1.

### 3.2. Test–Retest Reliability of Corticospinal Excitability

**Figure 2:**
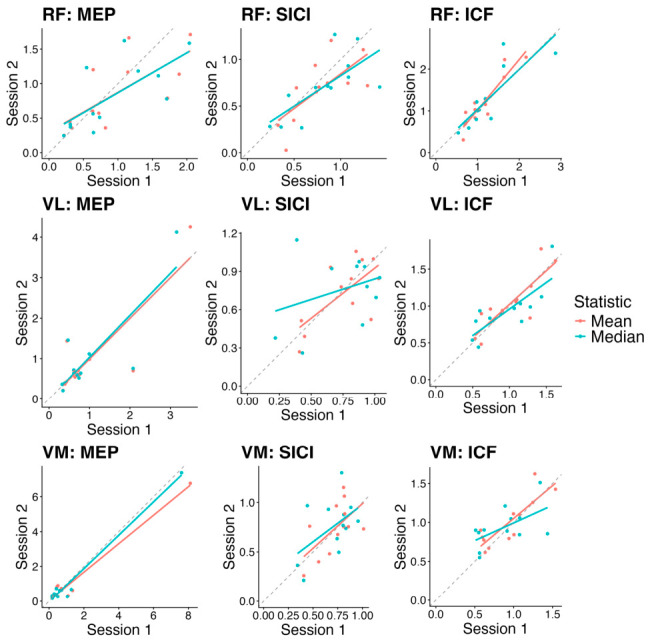
Scatterplots of repeated corticospinal and intracortical outcomes. RF: rectus femoris, VL: vastus lateralis, VM: vastus medialis, MEP: motor evoked potential, SICI: short-interval intracortical inhibition, ICF: intracortical facilitation.

The reliability of corticospinal excitability measures for the quadriceps muscles (i.e., MEP and AMT) is displayed in Table 2 and Figure 2. Each of the measured muscles demonstrated good to excellent reliability from the 10 MEP measures, and the AMT showed excellent reliability (Table 2).

### 3.3. Test–Retest Reliability of Intracortical Excitability

The reliability of the SICI and ICF in the quadriceps muscles is displayed in Table 3 and Figure 2. All muscles demonstrated moderate to excellent reliability, with only the mean measurement showing good reliability for SICI in the VL, whereas the median measurement shows moderate reliability. For ICF, only the median measurement of the VM showed reliability below good.

## 4. Discussion

This study found that, overall, the ICC for TMS measures of quadriceps corticospinal and intracortical excitability falls within the range of good to excellent reliability. The active motor threshold demonstrated excellent reliability in the RF. This supports previous data demonstrating that the AMT is reliable in the quadriceps muscle during a squat movement (ICC = 0.771) [9] but is a novel finding in the RF. With previous research demonstrating the reliability of the AMT in the upper limb [18,19], it follows that corticospinal excitability remains consistent in the lower limb too. However, this result shows that TMS is able to reliably measure corticospinal excitability in the quadriceps, despite complexities such as the depth of lower limb motor cortex representations and possibly interhemispheric interference due to the proximity of homologous cortical areas [14]. Given that research into cortical processes in lower limb injuries and conditions depends on TMS reliability, this research supports the credibility of relevant findings.

The motor evoked potential amplitude showed good or excellent reliability in all tested quadricep muscles. Whether the mean or median of each participant’s MEP trials were used did not impact reliability, with both measures resulting in good reliability for the RF and excellent reliability for the VM and VL. The RF is associated with smaller MEPs than the VM and VL [5], which may have led to greater relative variability and, therefore, lower reliability. However, this study demonstrated evidence that using the RF as the muscle of interest for TMS still provides reliable measures in the VL and VM. The reliability demonstrated in the present study supports the use of 120% AMT stimulation for the measurement of RF, VM and VL excitability using either mean or median measures of an individual’s trials.

The reliability of intracortical excitability did not appear to be as high as corticospinal excitability and varied between tested muscles. SICI showed good reliability in the RF for both mean and median measures but only moderate reliability in both measures for the VM. The VL demonstrated good reliability when using the mean; however, the ICC was not significant for the median measure. There was greater intra-individual MEP consistency for the RF, demonstrated by smaller standard deviations, which may have improved the reliability of SICI in this muscle. This could be attributed to using the RF to set the hotspot for the TMS assessment. Despite not targeting the VM and VL motor representation within the M1, reliability was good. This suggests that the lower reliability found for SICI could be attributed to true inter-session inconsistency in this measure, which has previously been found in active conditions of the finger [18]. Our results indicate that this variability can be partially mitigated by using the mean, and not median, of trials to represent an individual’s SICI (as both the VM and VL demonstrated higher reliability with this measure). However, this only alters the interpretation of the reliability for the VL. Using the mean measure of the active SICI in the VL still shows only moderate reliability, suggesting that future research may look to use additional trials beyond the 10 or assess reliability if the VL is the target muscle for the AMT and the ‘hotspot’.

All mean measures of ICF showed excellent reliability, with the median measure showing excellent reliability in the RF, good reliability in the VL and moderate reliability in the VM. Similar to SICI, these results support the use of the mean of trials in representing ICF of the quadriceps muscles. Previous research has shown that inhibition can be easier to elicit in distal muscles whilst facilitation is easier to elicit in proximal muscles [20]. This difference in the ability to elicit responses may explain why the reliability of ICF was generally better than SICI in the quadriceps. Future research may wish to replicate this methodology in a distal muscle of the lower limb (e.g., calf muscles) to determine if the reliability of intracortical excitability measures differ. Regarding research in the quadriceps, these results suggest that 10 trials are sufficient for reliability when using the mean measure of ICF.

### Limitations

The reliability of our TMS measures was only assessed during isometric contractions, which was due to the prominence of such contractions in the research. However, it is plausible that other contraction types, or even a rested state, would alter the reliability of corticospinal and intracortical measurements. Similarly, our study aimed to use the TMS methodology that is frequently reported in the literature, particularly regarding stimulation intensity, interstimulus intervals and alternative parameters. The TMS location was optimised for the RF and, although we believe there is sufficient evidence in our results to suggest this is sufficient for the VL and VM, the reliability of those muscle groups may be improved if these muscles were the primary target of the TMS. We also performed this study in healthy participants, and the results might not be able to be generalised to the broader population. Finally, as this study was nested within a randomised crossover trial, the sample size calculation was performed for the randomised crossover trial and not this exploratory reliability study.

## 5. Conclusions

This study established the test–retest reliability of corticospinal and intracortical excitability measures in the quadriceps. Taking the mean of an individual’s measures always resulted in similar or better reliability than the median, being determined as good or excellent in every measure of every muscle except for the SICI of the VM (which was moderate). With the previous reliability of TMS measures mostly investigating the upper limb, these results offer important context for neurophysiological research in the quadriceps.

## Figures and Tables

**Table 1:** Group mean (standard deviation) motor evoked potential values (mV), active motor threshold (% maximum stimulator output), short-interval intracortical inhibition and intracortical facilitation (% unconditioned MEP) in the lower limb.

TMS Variable	Muscle	Method	Session 1	Session 2
AMT	RF	-	44.2 (10.6)	44.4 (11.1)
MEP	RF	Mean	0.97 (0.63)	0.86 (0.509)
		Median	0.93 (0.61)	0.83 (0.50)
	VL	Mean	0.97 (0.92)	0.96 (1.08)
		Median	0.93 (0.84)	0.96 (1.05)
	VM	Mean	1.20 (2.20)	1.02 (1.83)
		Median	1.14 (2.06)	1.06 (2.00)
SICI	RF	Mean	0.78 (0.31)	0.68 (0.33)
		Median	0.82 (0.36)	0.71 (0.33)
	VL	Mean	0.75 (0.22)	0.73 (0.26)
		Median	0.75 (0.27)	0.76 (0.27)
	VM	Mean	0.73 (0.17)	0.74 (0.26)
		Median	0.70 (0.20)	0.75 (0.30)
ICF	RF	Mean	1.15 (0.46)	1.20 (0.60)
		Median	1.22 (0.62)	1.25 (0.71)
	VL	Mean	1.00 (0.36)	1.02 (0.38)
		Median	0.96 (0.37)	0.93 (0.34)
	VM	Mean	0.93 (0.32)	0.99 (0.31)
		Median	0.88 (0.32)	0.94 (0.26)

MEP: motor evoked potential, AMT: active motor threshold, SICI: short-interval intracortical inhibition, ICF: intracortical facilitation, RF: rectus femoris, VL: vastus lateralis, VM: vastus medialis, SD: standard deviation, IQR: inter-quartile range. Only group mean (SD) values are shown, calculated from either individual participant’s mean or median values.

**Table 2:** Reliability of quadriceps corticospinal and intracortical excitability.

TMS Variable	Muscle	Method	ICC	95%CI: Lower	95%CI: Upper	SEM	*p*-Value
MEP	RF	Mean	0.821	0.399	0.948	0.115	0.003 *
		Median	0.82	0.388	0.948	0.112	0.004 *
	VL	Mean	0.92	0.717	0.977	0.2	<0.001 *
		Median	0.903	0.657	0.972	0.19	<0.001 *
	VM	Mean	0.983	0.943	0.995	0.404	<0.001 *
		Median	0.993	0.977	0.998	0.406	<0.001 *
AMT	RF	-	0.991	0.971	0.997	2.17	<0.001 *

ICC: intraclass correlation coefficient, CI: confidence interval, SEM: standard error of the mean, MEP: motor evoked potential, AMT: active motor threshold, RF: rectus femoris, VL: vastus lateralis, VM: vastus medialis. This table shows lower and upper values of the 95% confidence interval of the ICC, as measured by participant’s mean and median values (n = 12). * *p* < 0.05.

**Table 3:** Reliability of quadriceps short-interval intracortical inhibition and intracortical facilitation.

TMS Variable	Muscle	Method	ICC	95%CI: Lower	95%CI: Upper	SEM	*p*-Value
SICI	RF	Mean	0.809	0.374	0.944	0.0653	0.004 *
		Median	0.813	0.386	0.945	0.0699	0.003 *
	VL	Mean	0.816	0.346	0.947	0.0484	0.005 *
		Median	0.513	−0.895	0.864	0.0533	0.138
	VM	Mean	0.724	−0.0121	0.922	0.0446	0.026 *
		Median	0.655	−0.228	0.899	0.0506	0.049 *
ICF	RF	Mean	0.941	0.803	0.983	0.107	<0.001 *
		Median	0.906	0.669	0.973	0.133	<0.001 *
	VL	Mean	0.924	0.735	0.978	0.0741	<0.001 *
		Median	0.888	0.608	0.968	0.0712	0.001 *
	VM	Mean	0.916	0.721	0.975	0.0632	<0.001 *
		Median	0.719	0.368	0.919	0.058	0.022 *

ICC: intraclass correlation coefficient, CI: confidence interval, SEM: standard error of the mean, SICI: short-interval intracortical inhibition, ICF: intracortical facilitation, RF: rectus femoris, VL: vastus lateralis, VM: vastus medialis. This table shows lower and upper values of the 95% confidence interval of the ICC, as measured by participants’ mean and median values (n = 12). * *p* < 0.05.

## Data Availability

Non-identifiable data relating to demographics, strength and neurophysiology are available upon reasonable request. The corresponding author can be contacted via the email address below to make this request.
